# Chaihu Shugan powder alleviates liver inflammation and hepatic steatosis in NAFLD mice: A network pharmacology study and *in vivo* experimental validation

**DOI:** 10.3389/fphar.2022.967623

**Published:** 2022-09-12

**Authors:** Sisi Lei, Shuai Zhao, Xiaoyan Huang, Yuchao Feng, Zhishang Li, Li Chen, Peiying Huang, Hansu Guan, Haobo Zhang, Qihua Wu, Bojun Chen

**Affiliations:** ^1^ Second Clinical Medical College, Guangzhou University of Chinese Medicine, Guangzhou, China; ^2^ Guangdong Provincial Key Laboratory of Research on Emergency in Traditional Chinese Medicine, Clinical Research Team of Prevention and Treatment of Cardiac Emergencies with Traditional Chinese Medicine, Guangzhou, China; ^3^ The Second Affiliated Hospital of Guangzhou University of Chinese Medicine, Guangzhou, China; ^4^ The Third Affiliated Hospital of Guangzhou University of Chinese Medicine, Guangzhou, China

**Keywords:** chaihu shugan powder, nonalcoholic fatty liver disease, inflammation, systems pharmacology, fatty acid synthesis, TNFα/TNFR1 pathway

## Abstract

**Background:** Nonalcoholic fatty liver disease (NAFLD) is the most common metabolic disease and is intertwined with cardiovascular disorders and diabetes. Chaihu Shugan powder (CSP) is a traditional Chinese medicine with a significant therapeutic effect on metabolic diseases, such as NAFLD. However, its pharmacological mechanisms remain to be elucidated.

**Methods:** The main compounds of CSP were measured using LC-MS/MS. A network pharmacology study was conducted on CSP. Its potential active ingredients were selected according to oral bioavailability, drug similarity indices, and phytochemical analysis. After obtaining the intersected genes between drug targets and disease-related targets, the component-disease-target network and protein-protein interaction analysis were visualized in Cytoscape. GO and KEGG enrichment analyses were performed using the Metascape database. Six-week-old male C57BL/6 mice fed a high-fat high-fructose diet for 16 weeks plus chronic immobilization stress for 2 weeks, an *in vivo* model, were administered CSP or saline intragastrically. Liver histology, triglyceride and cholesterol levels, ELISA, and RT-PCR were used to assess hepatic inflammation and steatosis. Immunohistochemistry and western blotting were performed to assess protein levels.

**Results:** A total of 130 potential target genes in CSP that act on NAFLD were identified through network pharmacology assays, including tumor necrosis factor (TNF), interleukin-6 (IL6), interleukin-1β (IL-1β), and peroxisome proliferator-activated receptor γ (PPARG). KEGG enrichment analysis showed that the main pathways were involved in inflammatory pathways, such as the TNF and NF-κB signaling pathways, and metabolism-related pathways, such as the MAPK, HIF-1, FoxO, and AMPK signaling pathways. The results *in vivo* showed that CSP ameliorated liver inflammation and inhibited hepatic fatty acid synthesis in the hepatocyte steatosis model. More specifically, CSP therapy significantly inhibited the expression of tumor necrosis factor α (TNFα), accompanied by a decrease in TNF receptor 1 (TNFR1) and the ligand availability of TNFR1.

**Conclusion:** Through the combination of network pharmacology and *in vivo* validation, this study elucidated the therapeutic effect of CSP on NAFLD, decreasing liver inflammation and inhibiting hepatic fatty acid synthesis. More specifically, the anti-inflammatory action of CSP was at least partially mediated by inhibiting the TNFα/TNFR1 signaling pathway.

## Introduction

Nonalcoholic fatty liver disease (NAFLD) encompasses a wide spectrum of diseases, ranging from simple steatosis and nonalcoholic steatohepatitis (NASH) to liver cirrhosis, which can progress to liver cancer, liver failure, and even death (Sheka et al., 2020). A recent study showed that the global prevalence of NAFLD has been estimated to be 25% (Golabi et al., 2021). Meanwhile, it has been estimated that patients with NAFLD have a 2-fold increase in the risk of cardiovascular disease (Younossi, 2019). The high incidence of NAFLD and its risk of cardiovascular events result in a huge economic burden to people worldwide (Mahjoubin-Tehran et al., 2021). Furthermore, there are no effective agents or standard pharmacologic therapies to treat NAFLD (Younossi et al., 2018). It is necessary to seek new therapeutic targets for NAFLD.

Chaihu Shugan powder (CSP), also called Chaihu Shugan San (CSS), is a traditional Chinese medicine (TCM) formula containing extracts of Radix Bupleuri (Chaihu in Chinese), Paeoniae Radix Alba (BaiShao in Chinese), Chuanxiong Rhizoma (Chuan Xiong in Chinese), Aurantii Fructus (ZhiQiao in Chinese), Citrus Reticulata (ChenPi in Chinese), Cyperi Rhizoma (XiangFu in Chinese) and licorice (GanCao in Chinese). It has been widely used in the treatment of depression and digestive diseases in China for four centuries (Wang et al., 2012). Quercetin, nobiletin, isorhamnetin, naringenin, kaempferol and stigmasterol are the main components of CSP. Pharmacological research has demonstrated that CSP and its components exhibit excellent anti-inflammatory, anti-hepatic steatosis, hypolipidemic and hypoglycemic effects (Dabeek and Marra, 2019; Li et al., 2019; Nie et al., 2020; Jayaraman et al., 2021). Our group also revealed that CSP could effectively improve glucose and lipid metabolic disorders in rats with metabolic syndrome (Lin et al., 2017). However, the curative effects and underlying mechanism of CSP on NAFLD remain unclear.

Meanwhile, there is evidence that inflammation plays a crucial role in NAFLD progression (Kim et al., 2016). Indeed, increased tumor necrosis factor α (TNFα) and its receptor TNF receptor 1 (TNFR1) levels in serum and liver have been found in the patients with NASH (Crespo et al., 2001; Rada et al., 2020). The TNF signaling pathway has been proven to not only be the key regulator of liver inflammation but also to have a significant influence on liver steatosis (Wandrer et al., 2020). Therefore, we speculated that the TNF signaling pathway may play a crucial role in the CSP treatment of NAFLD. However, as the greatest features of the TCM system, are multi components and targets, it has been an enormous challenge to uncover the association between herbal components and target diseases. Network pharmacology is an emerging strategy for the comprehensive study of TCM compounds based on multidisciplinary technologies, and it can reveal the core molecular targets and TCM components by a network visualization (Wang et al., 2021). In this study, we verified the effect of CSP on NAFLD mice. Both network pharmacology and *in vivo* experiments were used to unravel the underlying mechanisms.

## Materials and methods

### Screening of active compounds in CSP

The active compounds of CSP were collected from the Traditional Chinese Medicine Systems Pharmacology database (TCMSP, https://old.tcmsp-e.com/tcmsp.php, updated in 2020). The TCMSP is a specialized systems pharmacology database platform of Chinese traditional herbal medications, which contains the relationships among medications, targets, and diseases. Oral bioavailability (OB) is an important pharmacokinetic parameter for oral TCM, as it indicates the quantity of orally administered unchanged drugs that reach the systemic circulation. The Drug-likeness (DL) index refers to the structural similarity of compounds with known drugs, and it is widely utilized to assess the suitability of a component to be used as medication. Herein, the screening criteria in this research were an OB value of ≥30% and a DL value of ≥0.18.

### Potential targets intersection of CSP with disease

The potential targets of active compounds in CSP were retrieved from the TCMSP database and Swiss Target Prediction database (STP, http://swisstargetprediction.ch/, updated in 2022). The Uniport database (https://www.uniprot.org, updated in 2022) was used to convert protein names to their corresponding gene names.

### Collection of gene targets for NAFLD

NAFLD-related human genes were identified by searching the Gene Cards database (https://www. genecards.org/, version 5.9), Online Mendelian Inheritance in Man database (OMIM, http://omim.org/, updated in 2022), and Therapeutic Target Database (TTD, https://db.idrblab.net/ttd/, updated in 2021). “Non-alcoholic fatty liver disease” was used as the keyword.

### Components-genes network construction

The obtained drug targets were mapped to NAFLD disease targets to obtain a Venn diagram of the intersected gene symbols. After collecting the interactions of those targets from above, a complex information network was established. Finally, a visual analysis of the “Components-Genes Network” was conducted using Cytoscape 3.9.1 software. Cytoscape 3.9.1 is a robust bioinformatics software for visualizing molecular interaction networks.

### PPI network construction

The protein-protein interaction (PPI) network was obtained from the STRING database (https://string-db.org, version 11.5). The PPI network focused on finding hubs in which highly connected proteins were considered to play a crucial role between related ingredients and their probable targets. Afterward, all data were imported into Cytoscape 3.9.1 software to obtain PPI complex diagrams in a representable form to characterize the association between CSP and NAFLD intersection genes. The color and size of nodes represent the degree of the targets.

### GO and KEGG pathway enrichment analysis

To further characterize the molecular mechanism of CSP in NAFLD, Gene Ontology (GO) enrichment analysis and Kyoto Encyclopedia of Genes and Genomes (KEGG) pathway enrichment were performed by the Metascape database (https://metascape.org/, updated in 2022), with *p* < 0.01, min overlap value 3 and min enrichment value 1.5.

### Preparation of CSP aqueous extract

The CSP crude herbs were obtained pharmacy from Guang Dong Provincial Hospital of Chinese Medicine. All of these herbal medicines conformed to the requirements of the Chinese Pharmacopoeia 2020 edition and are in strict accordance with national execution standards. The dosage proportions of Radix Bupleuri (No.2106003, Shanxi, China; 5 g), Paeoniae Radix Alba (No.YPB1E0001, Anhui, China; 10 g), Chuanxiong Rhizoma (No.YPB0L0001, Sichuan, China; 5 g), Aurantii Fructus (No.210502, Jiangxi, China; 15 g), Citrus Reticulata (No. 210600521, Guangdong, China; 10 g), Cyperi Rhizoma (No. YPB1D0002, Anhui, China; 10 g) and licorice (No. YPB1D0002, Gansu, China; 10 g) was 6:6:4.5:4.5:4.5:4.5:1.5. To prepare the aqueous extract of CSP, all crude CSP herbs were decocted with an eightfold volume of distilled water twice, the first time for 1.5 h and the second time for 2 h. Then, the decoction was collected after filtration. Finally, the decoction was concentrated at 1.892 g/ml under reduced pressure using a rotary evaporator (IKA, Germany), and stored at 4°C.

### LC-MS/MS analysis

A Q-Exactive mass spectrometer (Thermo Fisher Scientific, Inc., San Jose, CA, United States) and an A Waters, ACQUITY UPLC HSS T3 (2.1 mm × 100 mm, 1.8 μm, Waters, Milford, MA, United States) column were used for LC separation. Gradient elution: acetonitrile/water (0.1% formic acid); flow rate of 0.2 ml/min. The column oven temperature was 45°C. The volume of the injection was 1 μL. Mass spectrometry analysis was performed using an electrospray ionization (ESI) source in positive and negative ion scanning mode. The parameters of the ESI source were as follows: sheath gas flow rate (arbitrary unit, Arb) was 35; aux gas (Arb) was 10; pray voltage was 3500 V; heated capillary temperature, 320°C; the analysis was carried out using a scan from 70 to 1,050 m/z. Scanning mode: full-scan mass spectra were set as follows: resolution, 70000; isolation window 70–1050 m/z, and data-dependent MS2 (dd-MS2) was set as follows: the resolution was 17500; normalized collision energy (NCE) was set at 20, 40, 60.

### Mice and experiments

Six-week-old male C57BL/6 mice were purchased from the Animal Research Laboratory of Guangdong Province (Guangzhou, China). The present study was conducted after approval by the Research Institute of the Animal Protection and Use Committee of Guangdong Provincial Hospital of Chinese Medicine [SCXK(Yue) 2021047]. After adaptive feeding, mice were randomly assigned to five groups (n = 6-7 per group): the control group (Control), the model group (Model), the low-dose of CSP intervention group (CSPL), the medium-dose of CSP intervention group (CSPM) and the high-dose of CSP intervention group (CSPH). The experimental protocols are as follows. Mice were fed a high-fat high-fructose diet (HFHFD, 40% kcal fat content, 20 kcal% fructose, and 2% cholesterol) for 16 weeks plus chronic immobilization stress (CIS, placed mice in a body-fit sized cylinder for 2 h per day for 2 weeks) to construct a NAFLD model. The mice in the control group were fed a normal diet only (13% kcal fat content). The daily dose of CSP decoction for human adults is 1.05 g/kg/d, and the equivalent dose in mice is 9.46 g/kg/d. The equivalent dose was selected as the medium dose; the high dose was doubling of the medium dose, while the low dose was half the medium dose.

### Metabolic assays and serum cytokine analyses

Mice were fasted for 6 h, and fasting blood-glucose (FBG) levels were examined by using a glucometer (Countour TS, Bayer, Parsipanny, NJ, United States). Blood lipid profiles, including total cholesterol (TC), triglycerides (TG), high-density lipoproteins (HDL-C) and low-density lipoproteins (LDL-C), and TC and TG contents in the liver were all measured using commercial kits (A111-2-1 for TC, A110-1-1 for TG, A112-2-1 for HDL-C and A113-2-1 for LDL-C, JianCheng Bioengineering Institute, Nanjing, China). The serum levels of alanine aminotransferase (ALT) and aspartate aminotransferase (AST) were measured by AST and ALT kits (C009-2-1 and C010-2-1, respectively, JianCheng Bioengineering Institute). The concentration of TNFα in the liver was examined by ELISA (EK1352, Signalway Antibody, Nanjing, China). All of the operations were performed strictly in accordance with the manufacturer’s protocols.

### Histology and immunohistochemical staining

Fresh liver samples were collected from the mice and fixed with 4% paraformaldehyde overnight and embedded in paraffin. Small sections from the liver (4 μm thick) were dewaxed with xylene, rehydrated in a graded ethanol series and stained with hematoxylin and eosin (H&E). The frozen liver samples were sliced (8 μm thick) and stained with Oil Red O (ORO) staining solution (G1620, Solarbio, Beijing, China). Nuclear factor κB (NF-κB) (1:300, 8242, CST, United States), sterol regulatory element-binding protein-1**(**SREBP-1**)** (1:300, sc-365513, Santa Cruz Biotechnology, United States) and peroxisome proliferator activated receptor (PPARγ) (1:200, sc-7273, Santa Cruz Biotechnology, United States) expression and localization in the liver sections of mice were investigated using immunohistochemical staining. After dewaxing with xylene and rehydration, the liver sections were stained using the EliVision™ plus immunohistochemical staining kits (MXB Biotechnologies, Fujian, China) following the kit’s instruction. The staining was visualized by using a microscope (Olympus, Tokyo, Japan).

### Quantitative real-time PCR

Total mRNA was isolated from fresh liver samples using RNAiso Plus (AKF0722A, Takara, Otsu, Shiga, Japan) and reverse-transcribed into cDNA using an Evo M-MLV RT kit (AG11728, Accurate Biology, Hunan, China) according to the manufacturer’s protocol. SYBR Green Master Mix (Rox Plus) (AG11718, Accurate Biology, Hunan, China) was applied to quantify PCR amplification. Relative mRNA expression levels were normalized to the β-actin expression level. Real-time PCR results were calculated using the 2^−ΔΔCt^ method. The primer pairs used in this study were synthesized by Shanghai General Biotech Co., Ltd., The primer information is listed in Supplemental Table S1.

### Western blotting analysis

Total protein samples were isolated from tissue samples using RIPA lysis buffer (P0013B, Beyotime, Shanghai, China) containing both PMSF and phosphatase inhibitors for western blot analyses. A BCA Protein Assay Kit (23225, Thermo Fisher Scientific, Rockford, IL, United States) was used to determine the protein concentration. The protein samples were separated on the 10% or 12% SDS-PAGE gels, and then transferred to a polyvinylidene fluoride (PVDF, Millipore, MA, United States) membranes. After blocking with 5% skim milk, the PVDF membranes were incubated overnight at 4°C with the following primary antibodies: anti-TNFR1 (1:1000, 21574-1-AP, Proteintech, China), anti-phospho-NF-κB p65(Ser536) (1:1000, 8324, CST), anti-IL1β (1:1000, ab6722, Abcam, United States), IL6 (1:1000, bs-6309R, Bioss), iNOS (1:1000, bs-0162R, Bioss), anti-TNFα (1:1000, bsm-33207M, Bioss), anti-SREBP-1 (1:1000, sc-365513, Santa Cruz Biotechnology), anti-PPARγ (1:1000, bsm-4590R, Bioss) and β-actin (1:5000, AC026, ABclonal, China). After that, the PVDF membranes were incubated with the corresponding secondary antibodies. An ECL kit (Tanon, Shanghai, China) was used to visualize the chemiluminescence signals. Protein expression levels were quantified with ImageJ software. The housekeeping protein β-actin was utilized as loading control.

### Statistical analysis

All data in this study are expressed as the mean ± s.d. One-way analysis of variance (ANOVA) followed by LSD or Dunnett’s T3 post-hoc test was applied for the results using the SPSS 25.0 statistical software package. Data for graphing were processed with GraphPad Prism 8.4.3 software. A *p* value < 0.05 was considered significant.

## Results

### Bioactive compounds in CSP

The phytochemical composition of CSP was evaluated using LC-MS/MS. The total positive and negative ion chromatograms of CSP aqueous extracts, such as quercetin, kaempferol, naringenin, isorhamnetin, and nobiletin, are shown in Supplementary Figure S1 and Supplementary Table S2. This mass spectrometry closely replicated previous findings (Han et al., 2021).

### Compound-gene network of CSP and NAFLD

**FIGURE 1:**
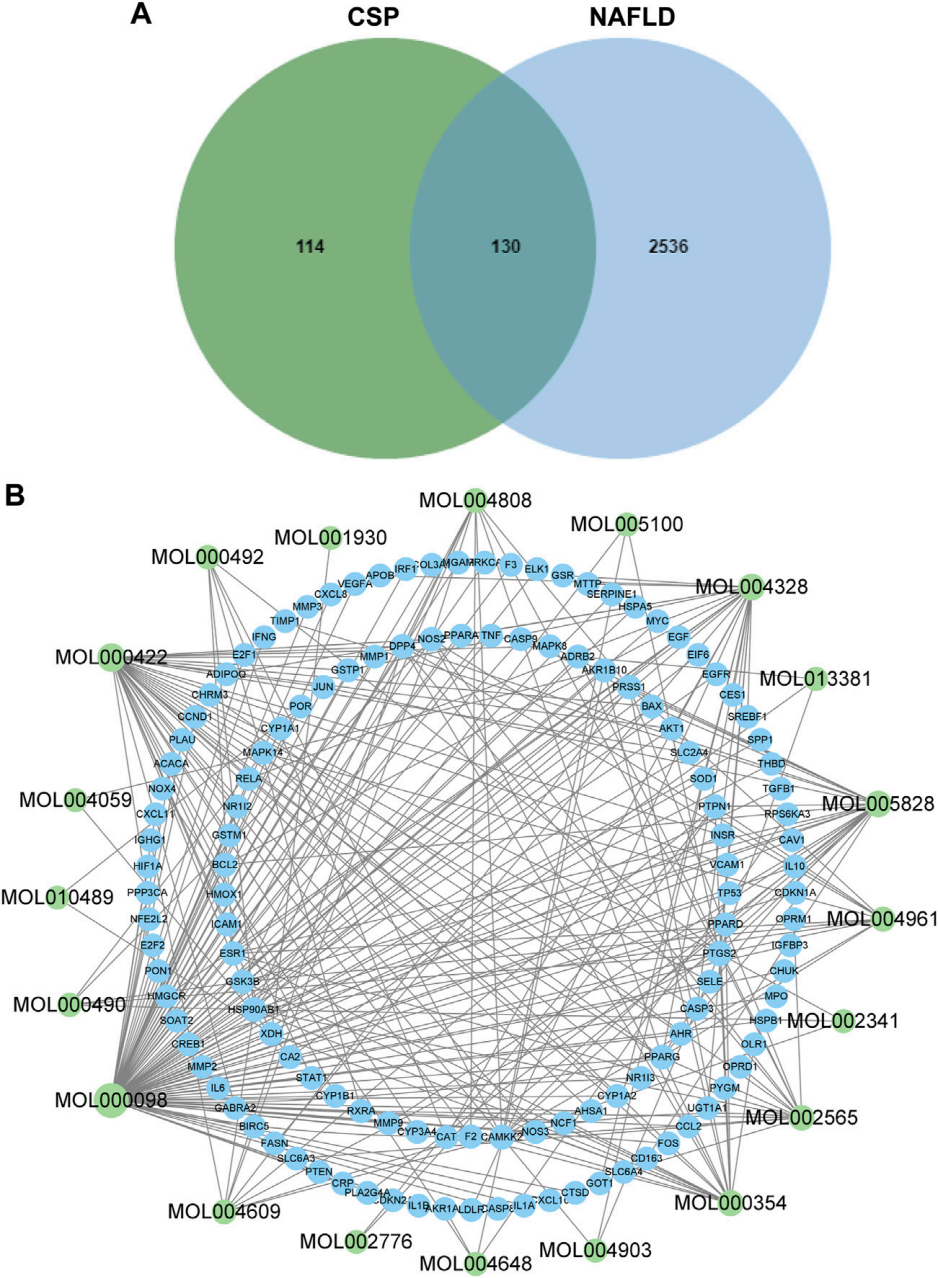
Components-Genes Network of CSP and NAFLD. **(A)** Overlaps between NAFLD genes and CSP-related genes; **(B)** The drug-bioactive ingredient target-disease network of CSP acting on NAFLD. Green nodes represent the compounds; blue nodes represent target genes.

A total of 139 active compounds in CSP were selected from the TCMSP database according to our search criteria (Supplementary Table S3). After taking an intersection of the results of LC-MS/MS analysis, a total of 21 main compounds were collected (Table 1). Meanwhile, the TCMSP database and SwissTarget Prediction database were used to collect the prediction targets of the main compounds of CSP. A total of 244 predicted target genes were acquired after removing duplicated targets (Supplementary Table S4). We also identified a total of 2762 therapeutic targets for NAFLD in 3 different databases (1512 from GeneCard, 1243 from OMIM, and 7 from TTD) (Supplementary Table S5). Once the superfluous targets were eliminated, a total of 2666 known therapeutic targets for NAFLD remained. Ultimately, we obtained 130 overlapping targets that could be instrumental in treating NAFLD using the main compounds of CSP (Figure 1A and Supplementary Table S6). The “Compounds-Genes Network” was constructed using Cytoscape software to elucidate the likely mechanism of CSP acting on NAFLD (Figure 1B). According to network topological parameters, quercetin (MOL000098, degree = 97) exhibited the highest correlation with the target genes of NAFLD, and the rest were kaempferol (MOL000422, degree = 42), naringenin (MOL004328, degree = 27), nobiletin (MOL005828, degree = 25), isorhamnetin (MOL000354, degree = 23), and so on.

**TABLE 1:** The main compounds in CSP (OB ≥ 30% and DL ≥ 0.18).

Mol ID	Molecule name	OB (%)	DL	Herb	Targets
MOL000354	isorhamnetin	49.6	0.31	ChaiHu, XiangFu, GanCao	37
MOL000098	quercetin	46.43	0.28	ChaiHu, XiangFu, GanCao	154
MOL001930	benzoyl paeoniflorin	31.27	0.75	BaiShao	2
MOL000492	(+)-catechin	54.83	0.24	BaiShao	11
MOL000490	petunidin	30.05	0.31	ChaiHu	8
MOL002776	Baicalin	40.12	0.75	ChaiHu	2
MOL004609	Areapillin	48.96	0.41	ChaiHu	17
MOL004648	Troxerutin	31.6	0.28	ChaiHu	15
MOL005100	5,7-dihydroxy-2-(3-hydroxy-4-methoxyphenyl) chroman-4-one	47.74	0.27	ChenPi	10
MOL000211	Mairin	55.38	0.78	BaiShao, GanCao	1
MOL004328	naringenin	59.29	0.21	ZhiQiao, ChenPi, GanCao	37
MOL004808	glyasperin B	65.22	0.44	GanCao	21
MOL004903	liquiritin	65.69	0.74	GanCao	6
MOL002565	Medicarpin	49.22	0.34	GanCao	34
MOL004961	Quercetin der	46.45	0.33	GanCao	17
MOL005828	nobiletin	61.67	0.52	ZhiQiao, ChenPi	35
MOL004059	khellol glucoside	74.96	0.72	XiangFu	15
MOL010489	Resivit	30.84	0.27	XiangFu	4
MOL013381	Marmin	38.23	0.31	ZhiQiao	4
MOL002341	Hesperetin	70.31	0.27	ZhiQiao	9
MOL000422	kaempferol	41.88	0.24	ChaiHu, BaiShao, XiangFu, GanCao	63

### PPI analysis

**FIGURE 2:**
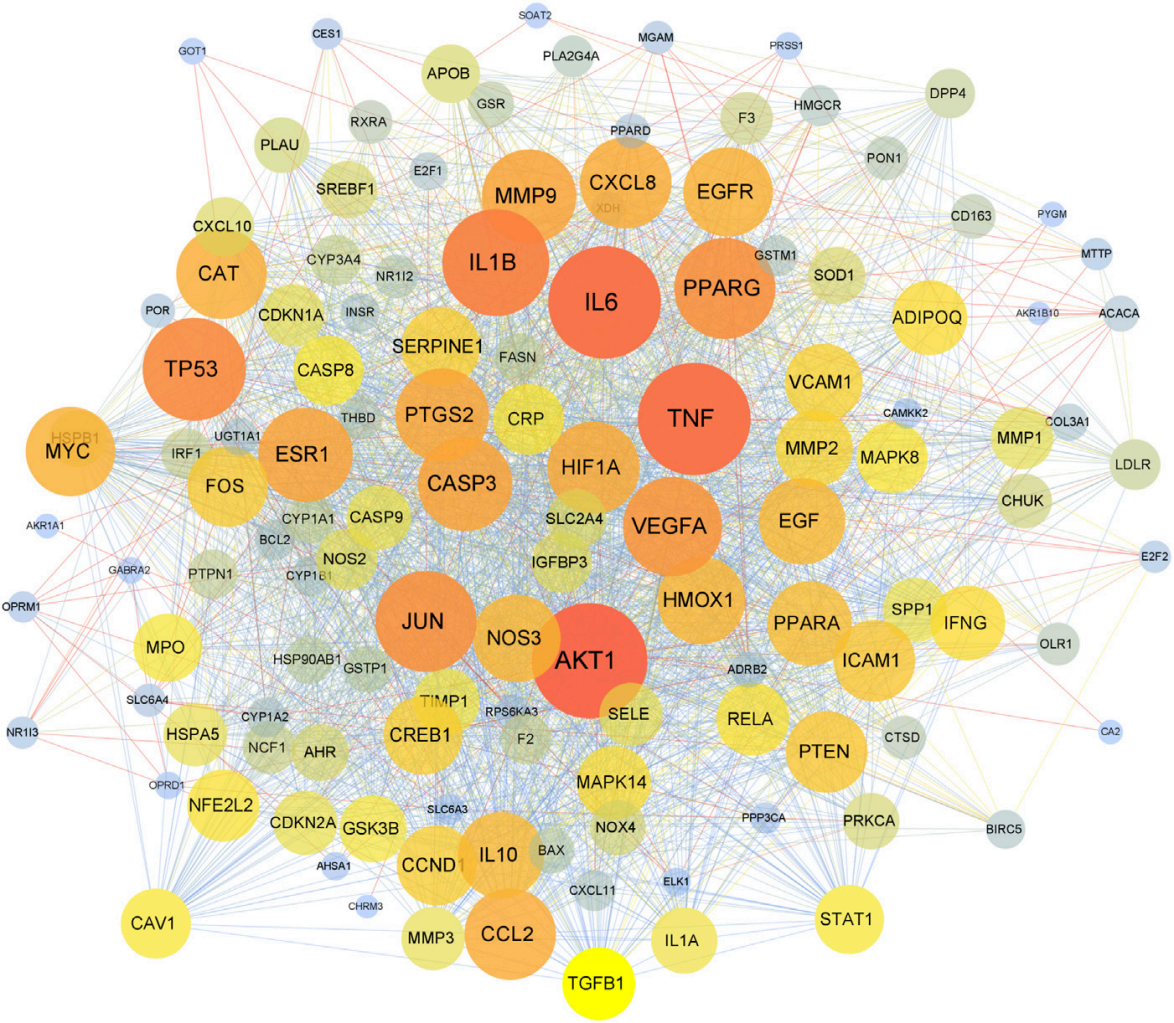
The PPI network. The nodes represent overlapping proteins, and the edges represent the interactions among the proteins. The color and size of nodes represent the degree of the targets.

The 130 overlapping targets were uploaded to the STRING database to obtain information on predicted interactions. Then, the STRING data were imported into Cytoscape to devise a PPI network (Figure 2). There were 130 protein nodes and 2365 edges remaining in the PPI network after removing targets that lack protein structure and have no interaction with other proteins. Proteins are connected with other proteins; as proteins exhibit a higher degree, they play a more critical role in the central correlation. The top 10 proteins ranked by degree value were *TNF, IL6, IL1B, AKT1, PPARG, JUN, TP53, VEGFA, MMP9,* and *CASP3.*

### GO and pathway enrichment analysis

**FIGURE 3:**
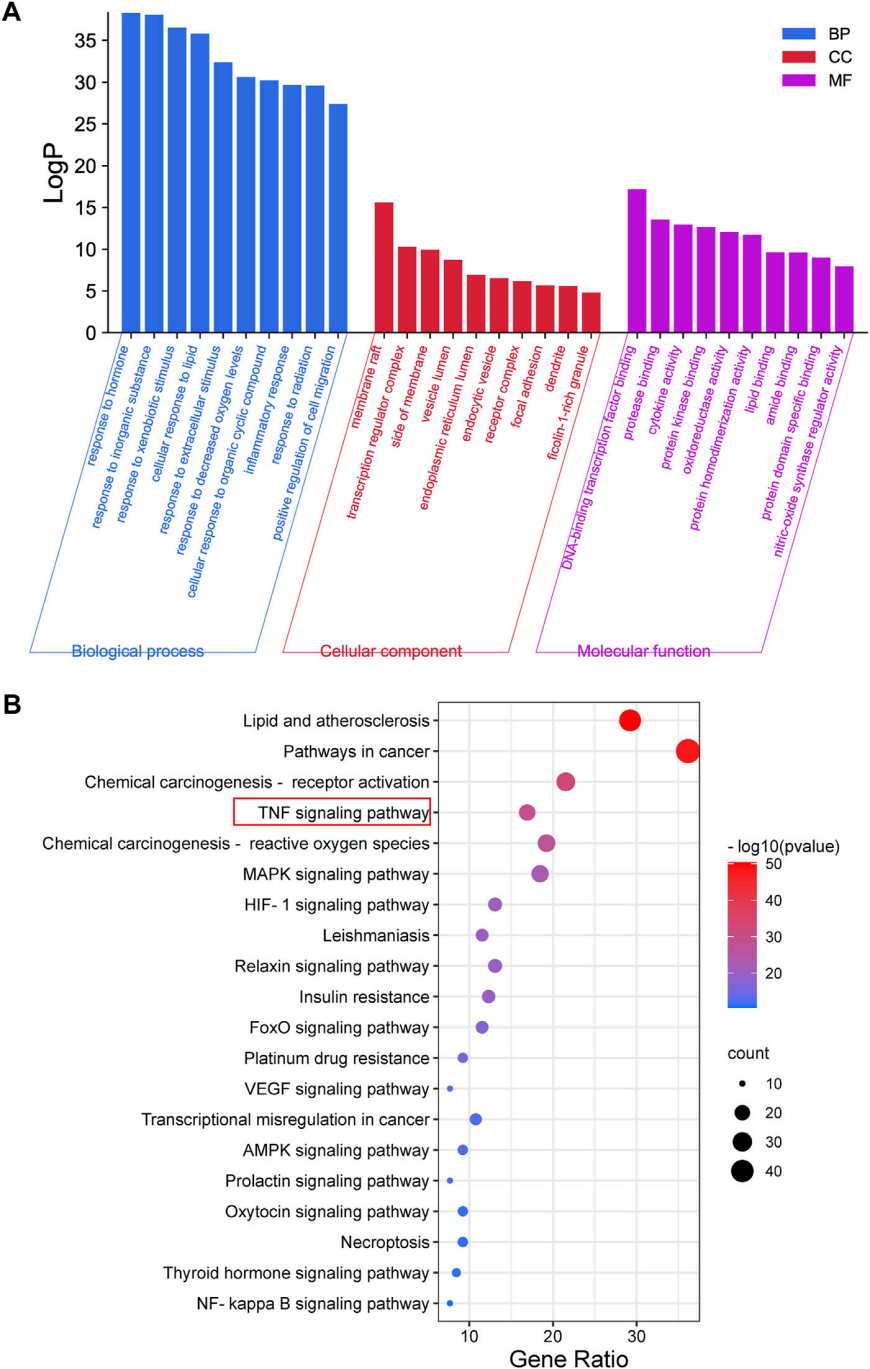
GO and Pathway Enrichment Analysis. **(A)** The GO enrichment analysis for overlapping targets between CSP and NAFLD. **(B)** KEGG pathway enrichment analysis of overlapping targets between CSP and NAFLD.

GO and pathway enrichment analyses of 130 overlapping targets were conducted using the Metascape Database (Supplementary Table S7). The GO results showed 1633 biological process (BP), 92 cellular component (CC), and 146 molecular function (MF) terms in total. The top 10 BP, CC, and MF terms are shown in Figure 3A. The terms in BP include response to lipid, inflammation, hormone, etc. The terms in CC include membrane raft, transcription regulator complex, side of the membrane, etc. The MF terms included DNA-binding transcription factor binding, protease binding, cytokine activity, etc. Meanwhile, a total of 193 enriched pathways were selected by KEGG analysis. The top 20 significantly enriched pathways are shown in Figure 3B. The results showed that overlapping targets were mainly enriched in lipid and atherosclerosis pathways, pathways in cancer and chemical carcinogenesis-receptor activation, and the TNF signaling pathway. The results of GO and pathway enrichment analyses indicated that the mechanism of CSP in NAFLD may be highly related to the TNF signaling pathway.

### CSP ameliorated liver lipid accumulation in NAFLD mice

**FIGURE 4:**
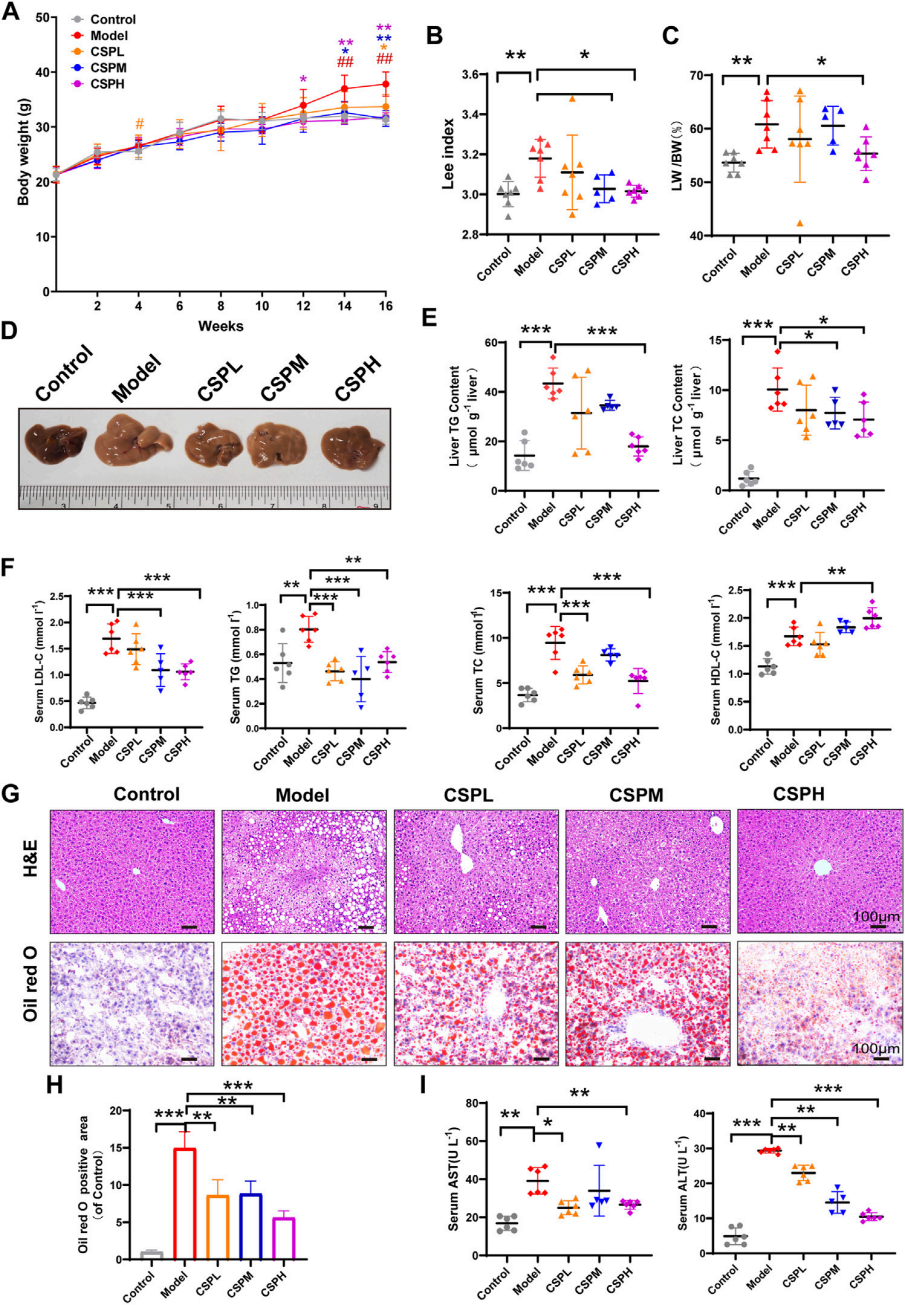
CSP ameliorated obesity and liver lipid accumulation in mice fed a HFHFD diet and CIS. **(A–C)** The mouse body weights at 0, 2, 4, 6, 8, 12, 14, and 16 weeks and the Lee index and liver-to-body weight ratio at 16 weeks, means ± s.d. ^#^ *p* < 0.05, ^##^ *p* < 0.01, *p < 0.05, **p < 0.01, ^#^ vs. the control group, * vs. the model group, n = 5-7 samples per group; **(D)** Representative images of mouse livers after 16 weeks of treatment. **(E,F)** The lipid profiles in the liver and serum of mice after 16 weeks of treatment; **(I)** Serum AST and ALT concentrations after 16 weeks of treatment; **(G,H)** Representative images of H&E-stained sections and Oil Red O-stained sections of the liver at 16 weeks (scale bar: 100 μm). Data in **(E–I)** are presented as the means ± s.d.*p < 0.05, **p < 0.01, ***p < 0.001, **(E,F,I)** n = 5-6 samples per group, **(H)** n = 3 samples per group.

Although, HFHFD treatment could induce hepatic steatosis in mice. Recent studies discovered that combination treatment of HFHFD plus CIS could promote chronic inflammation in the liver and faster development of NAFLD (Corona-Pérez et al., 2017). Therefore, we used this combination treatment to construct the NAFLD model in this study. As shown in Figure 4A, in response to the combination treatment of HFHFD plus CIS, the body weight of mice in the model group increased markedly during the period between 14 and 16 weeks compared with the control group. Meanwhile, CSP intervention inhibited the body weight increase caused by the combination treatment of HFHFD plus CIS in a concentration-dependent manner, as well as the Lee index **(** Figure 4B **)**. As illustrated in **(** Figures 4C,D,G,H **)**, the mice of the model group also exhibited a prominent fatty liver phenotype featuring a pale and enlarged liver, higher liver-to-body weight ratio, increased fat vacuoles shown in H&E staining, and increased red lipid droplets shown in Oil red O staining. The lipid profiles in the liver and in the serum and liver enzyme levels were all elevated in the model group (Figures 4E,F,I). In contrast, after 16 weeks of CSP treatment, the liver size, liver-to-body weight ratio, fat vacuoles, and red lipid droplets were distinctly decreased (Figures 4C,D,G,H). The lipid profiles in liver and blood and liver enzymes were also considerably decreased (Figures 4E,F,I). However, higher HDL-C levels were observed in both the model group and the CSPH group than in the control group. All of these results indicated that CSP had a positive effect on the development of NAFLD in mice fed a HFHFD plus CIS. CSP also decreased FBG levels in mice fed a HFHFD plus CIS (Supplementary Figure S2).

### CSP inhibits inflammatory cytokine and hepatic fatty acid synthesis *in vivo*

**FIGURE 5:**
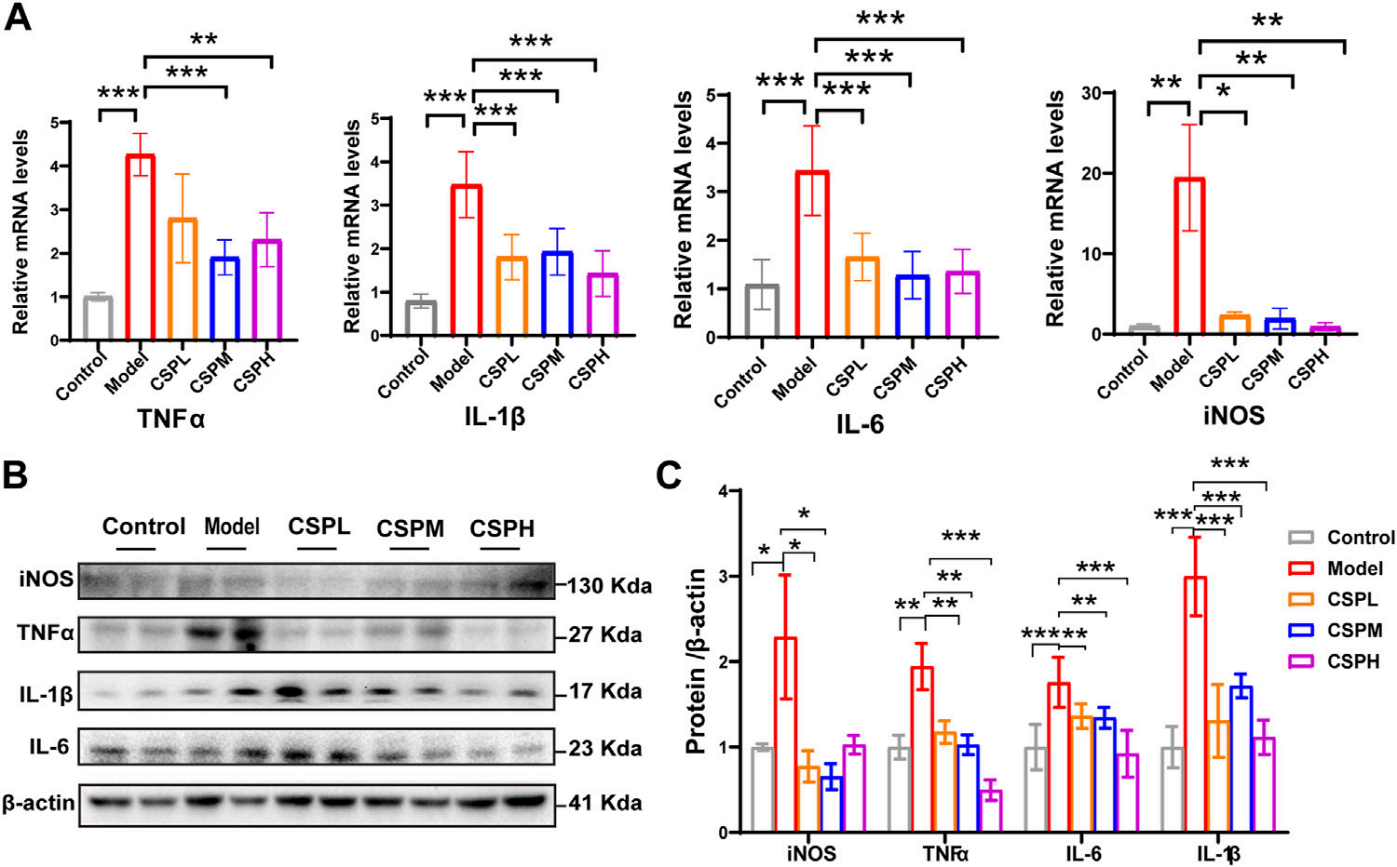
CSP inhibits inflammatory cytokines in the liver of a mouse model of NAFLD. **(A)** TNFα, IL-1β, IL-6, and iNOS expression levels in the liver measured by RT-PCR analyses; **(B)** Western blot analyses of TNFα, IL-1β, IL-6 and iNOS with β-actin as a loading control; **(C)** Densitometric analyses of band intensities normalized to β-actin. Data **(A,C)** are presented as the means ± s.d. *p < 0.05, **p < 0.01, ***p < 0.001, n = 5-6 samples per group.

Inflammation is an important factor affecting the progression of NAFLD. The level of inflammation in NAFLD mouse liver tissue in response to CSP was examined using the RNA and protein levels of inflammatory cytokines, including TNFα, interleukin-6 (IL6), interleukin-1β (IL-1β), and inducible nitric oxide synthase (iNOS). Compared with the control group in liver tissue, TNFα, IL-1β, IL-6, and iNOS in the liver of the model group were all significantly increased at the mRNA level (Figure 5A). In contrast, the mRNA levels of hepatic TNFα, IL-1β, IL-6, and iNOS were decreased after CSP intervention (Figure 5A). Moreover, the protein levels of TNFα, IL-1β, IL-6, and iNOS exhibited a similar trend to the mRNA levels (Figures 5B,C).

**FIGURE 6:**
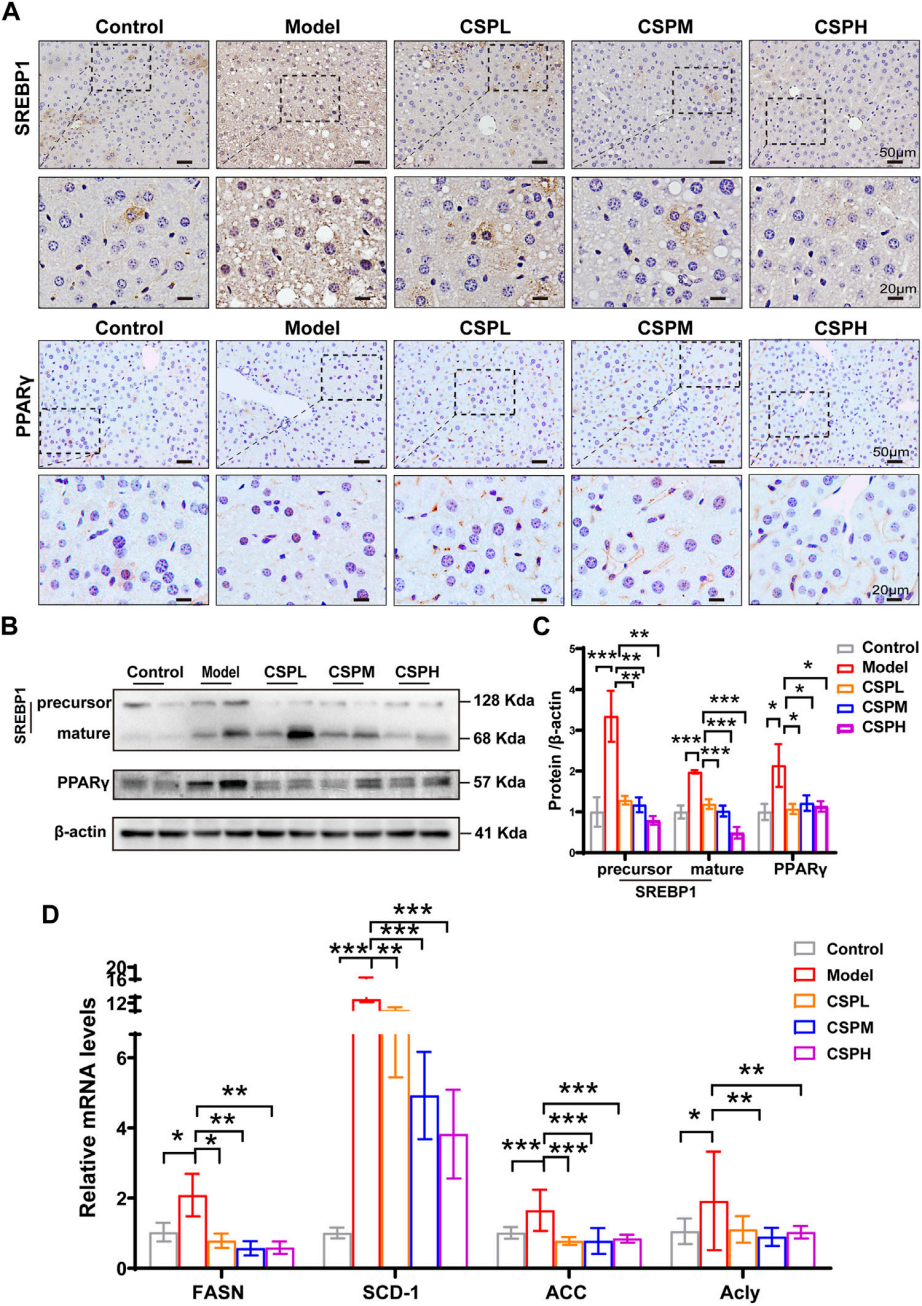
CSP treatment inhibited hepatic fatty acid synthesis in mice fed a HFHFD plus CIS. **(A)** Representative images of immunohistochemical staining of SREBP-1 and PPARγ in the liver sections of mice at 16 weeks (scale bar: 50 μm and 20 μm). **(B)** The protein expression levels of precursor SREBP-1, mature SREBP-1, and PPARγ measured in the livers of mice at 16 weeks. **(C)** SCD-1, FASN, ACC and Acly expression levels in the liver from mice at 16 weeks measured by RT-PCR analyses. Data **(C,D)** are presented as the means ± s.d. *p < 0.05, **p < 0.01, ***p < 0.001, n = 5-6 samples per group.

SREBP-1 and PPARγ, both key transcriptional regulators in hepatic lipogenesis, were subjected to immunochemical staining of the liver in this study. Compared with the control group, a marked increase in the nuclear expression of SREBP-1 and PPARγ in the liver of the model mice and CSP treatment could reverse this tendency (Figure 6A). The protein expression exhibited similar trends to the immunochemical staining (Figures 6B,C). This was consistent with the prediction that PPARG and SREBF-1 have important roles in the PPI network of CSP in NAFLD. We also tested the mRNA expression levels of fatty acid synthases, including stearoyl-CoA desaturase 1 (SCD-1), fatty acid synthase (FASN), acetyl-CoA carboxylase (ACC), and ATP-citrate lyase (Acly). The mRNA expression levels of these fatty acid synthases in the CSP group were all tremendously decreased compared to those in untreated mice in the model group (Figure 6D). Collectively, CSP treatment decreased liver inflammation and suppressed hepatic fatty acid synthesis in mice fed a HFHFD plus CIS.

### CSP Inhibits the Expression of TNFα/TNFR1 and Lowers the Ligand Availability of TNFR1 *in vivo*

**FIGURE 7:**
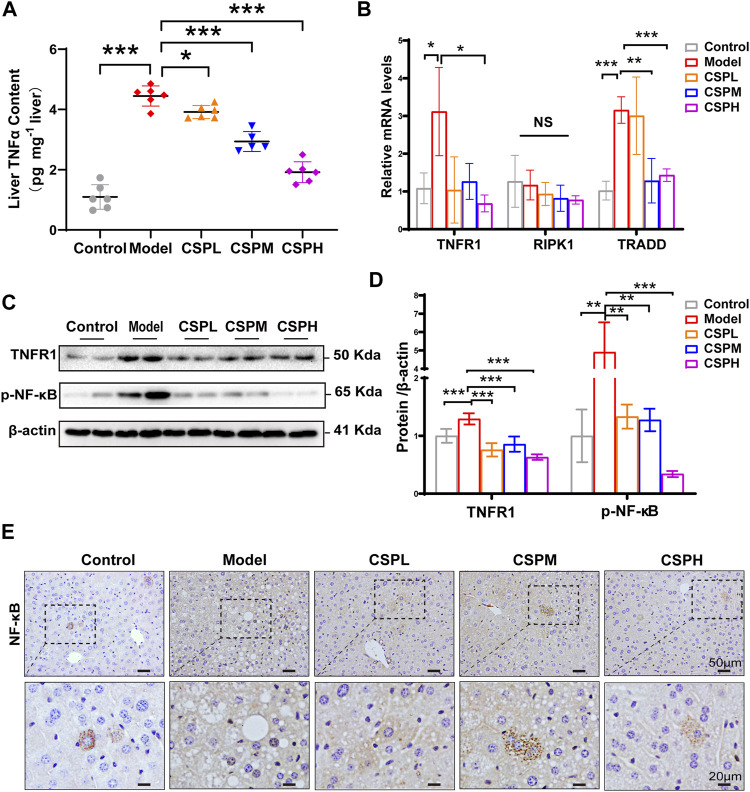
CSP inhibited the expression of TNFα and TNFR1 and lowered the ligand availability of TNFR1 in the livers of mice with HFHFD plus CIS. **(A)** TNFα content in liver assessed by ELSA assay; **(B)** The mRNA expression levels of TNFR1, RIPK1, and TRADD in the liver measured by RT-PCR analyses; **(C)** and **(D)** The protein expression levels of p-NF-κB and TNFR1 in the TNF signaling measured in the liver; **(E)** Representative images of immunohistochemical staining of NF-κB in liver sections (scale bar: 50 and 20 μm). Data **(A,B–D)** are presented as the means ± s.d. NS = no significant difference, *p < 0.05, **p < 0.01, ***p < 0.001, n = 5-6 samples per group.

The TNF signaling pathway is one of the most well-studied inflammatory signaling pathways. In addition, the results of the KEGG pathway enrichment analysis indicated that the overlapping targets of CSP in NAFLD were significantly enriched in the TNF signaling pathway. To determine whether CSP treatment ameliorated liver inflammation by regulating the TNF signaling pathway. The liver TNFα contents were examined using an ELSA kit, and the results showed that TNFα secretion in the liver of the model group was markedly increased compared with that in the control group and decreased after CSP treatment (Figure 7A). TNF signaling-related factors, including TNFR1, its adaptor protein receptor-interacting serine-threonine kinase 1 (RIPK1), TNFR-associated death domain (TRADD), and its downstream transcription factor (NF-κB), were also assessed. Mice in the model group had higher hepatic mRNA expression of TNFR1 and TRADD than mice in the control group (Figure 7B). In contrast, the hepatic mRNA levels of TNFR1 and TRADD in the livers of the CSP groups were significantly reduced (Figure 7B). The protein expression level of TNFR1 in these groups was consistent with its mRNA expression (Figures 7C,D). In addition, compared with the model group, the protein levels of p-NF-κB exhibited a significant reduction in the three-dose CSP group (Figures 7C,D). Immunohistochemical staining in the liver of the CSP intervention groups showed that nuclear NF-κB was significantly reduced compared with that in the model group (Figure 7E). However, the mRNA levels of hepatic RIPK1 were unaffected by both the model group and the CSP groups (Figure 7B). These results indicated that lowering TNFα/TNFR1 expression and ligand availability was required for the therapeutic effect of CSP on NAFLD.

## Discussion

The present study demonstrated a novel mechanism of CSP that alleviates NAFLD through network pharmacology and experimental validation. By comprehensive analysis of the network of components-targets-pathway and mass spectrometry, among the 21 active components of CSP, quercetin was the most significant component, followed by kaempferol, naringenin, nobiletin, and isorhamnetin. The PPI network exhibited the priority of interactions between 130 drug-disease overlapping targets. Target proteins such as *TNF, IL6, IL1B, AKT1, PPARG, JUN, and TP53* have shown a more important position and have close interactions between targets. A previous study found that quercetin could markedly exude and increase the degree of inflammation in the lamina propria of the nasal cavity, in parallel with reducing the secretion of TNFα and IL6 (Tiboc-Schnell et al., 2020). Reportedly, quercetin and kaempferol were also found to be effective against the activation of PPARγ to combat the deleterious effect of thiazolidinediones (Lokhande et al., 2020). In addition, isorhamnetin and nobiletin were found to attenuate inflammation and endothelial cell apoptosis (Li et al., 2016; Bunbupha et al., 2020). These past studies verified the effects of active ingredients in CSP on anti-inflammation, modulating PPARγ activity, and anti-apoptosis, which is consistent with our prediction results of the network.

Meanwhile, the response to lipids, inflammation and hormones was highly enriched in biological process terms of GO enrichment. The results of the *in vivo* experiment confirmed that CSP therapy ameliorates liver inflammation by inhibiting both the mRNA and protein levels of inflammatory factors, including TNFα, IL-1β, IL-6, and iNOS in the livers of mice with NAFLD. Here, the influence of CSP on hepatic fatty acid synthesis was investigated by measuring the protein levels and activation of SREBP-1 and PPARγ. SREBP-1c and SREBP-1a, two SREBP-1 isoforms, are all pivotal regulators of cellular cholesterol and fatty acid homeostasis (Zhao et al., 2021). SREBP-1c, abundant in the liver, primarily activates fatty acid synthesis (Moon et al., 2012). SREBP-1a is abundant in growing cells except for the liver and activates cholesterol and fatty acid synthesis (Knebel et al., 2018). PPARγ, a critical factor in the regulation of hepatic lipogenesis, is also reported to be robustly induced in the livers of patients, as well as in preclinical models of NAFLD (Hasenfuss et al., 2014; Platko et al., 2019). The immunohistochemistry and Western blot results in this study showed that after CSP gavage, the protein levels and nuclear localization of SREBP-1 and PPARγ in the liver were reduced compared with those in the model group. Genes involved in *de novo* lipogenesis, such as SCD-1, ACC, FASN, and Acly, are regulated by SREBP-1 and PPARγ (Wada et al., 2008; Shimano and Sato, 2017) were also detected in this study, and all of them showed a similar trend.

To investigate the upstream regulation of inflammation, enrichment analysis of KEGG pathways was carried out for key targets. The TNF signaling pathway was significantly enriched. TNFα, an inflammatory cytokine in liver tissues, was confirmed as a critical driver of every process of NAFLD (Yang et al., 2020). In addition, the present *in vivo* study also discovered a higher secretion of TNFα in the liver of the model group than in the control group, which was significantly decreased after CSP treatment. As a key inflammatory activator, TNFα signal transduction is mediated by TNF-receptor-1 (TNFR1) and TNF-receptor-2 (TNFR2) (Wang et al., 2020). TNFR1 is expressed on most cell types, whereas TNFR2 expression is found primarily on endothelial and immune cells (Kim et al., 2013). Indeed, the present *in vivo* study found that CSP therapy significantly decreased the protein and mRNA levels of TNFR1 in the liver tissue of mice with NAFLD. In addition, upon TNFα binding to TNFR1, the cytoplasmic domain of TNFR1 containing TRADD and RIPK1 is triggered by trimerization, which is followed by the activation of NF-κB and eventually leads to chronic inflammation in the liver by upregulating inflammatory cytokines such as TNFα, IL1β, IL6 and iNOS (Ermolaeva et al., 2008; Li et al., 2017; Van Quickelberghe et al., 2018). It is worth pointing out that in the results of this study, the expression of TNFα and TNFR1 in the liver of the model group increased, as did TRADD mRNA. In contrast, the mRNA level of TRADD was significantly decreased in the liver tissue of the CSP groups. These data indicate that CSP could inhibit the activation of NF-κB and attenuate liver inflammation in mice with HFHFD plus CIS by inhibiting the TNFα/TNFR1 signaling pathway. In addition, there is increasing evidence that TNFα/TNFR1-mediated pathways create a pleiotropic microenvironment that plays a critical role in inflammation in the liver, hepatic steatosis, and fibrogenesis and thus in NAFLD progression (Tarrats et al., 2011; Gong et al., 2014). TNFα treatment could increase the formation of lipid droplets following upregulated expression of FAS and activation of SREBP-1 (Choi et al., 2012). Meanwhile, inhibition of TNFR1 could significantly reduce SREBP-1 activation and downstream targets of lipogenesis, such as FAS and SCD1 (Wandrer et al., 2020). Intriguingly, in this study, compared with the model group, lower activation of SREBP-1 and decreased downstream targets of lipogenesis were detected in liver tissues from the CSP groups. These data therefore assume an important role of the TNFα/TNFR1 pathway in the activation of SREBP-1 and hepatic fatty acid synthesis in mice with NAFLD. However, the detailed pharmacological mechanisms still need to be further studied. Overall, the results of this study demonstrated that CSP could attenuate hepatic steatosis and decrease liver inflammation via the TNFα/TNFR1 signaling pathway in mice with HFHFD plus CIS.

## Conclusion

In conclusion, this study provided valuable novel insights to advance our comprehension of the mechanism underlying the action of CSP on NAFLD. Using network pharmacology and *in vivo* experiments, this research revealed that CSP may have the capacity to effectively alleviate hepatic steatosis in the progression of NAFLD and inhibit the inflammatory response through the TNFα/TNFR1 signaling pathway, which also paves the way for the specific molecular mechanism of CSP in NAFLD. Additionally, the network pharmacology framework explained in this study has the potential to provide fresh insight into the treatment of NAFLD with TCM.

## Data Availability

The datasets presented in this study can be found in online repositories. The names of the repository/repositories and accession numbers can be found in the article/Supplementary Material.
