# Supplementary figures and images for: Chaihu Shugan powder alleviates liver inflammation and hepatic steatosis in NAFLD mice: A network pharmacology study and in vivo experimental validation
Source: Front Pharmacol. 2022 Sep 12;13:967623. doi: 10.3389/fphar.2022.967623 (PMC9512055; doi:10.3389/fphar.2022.967623)

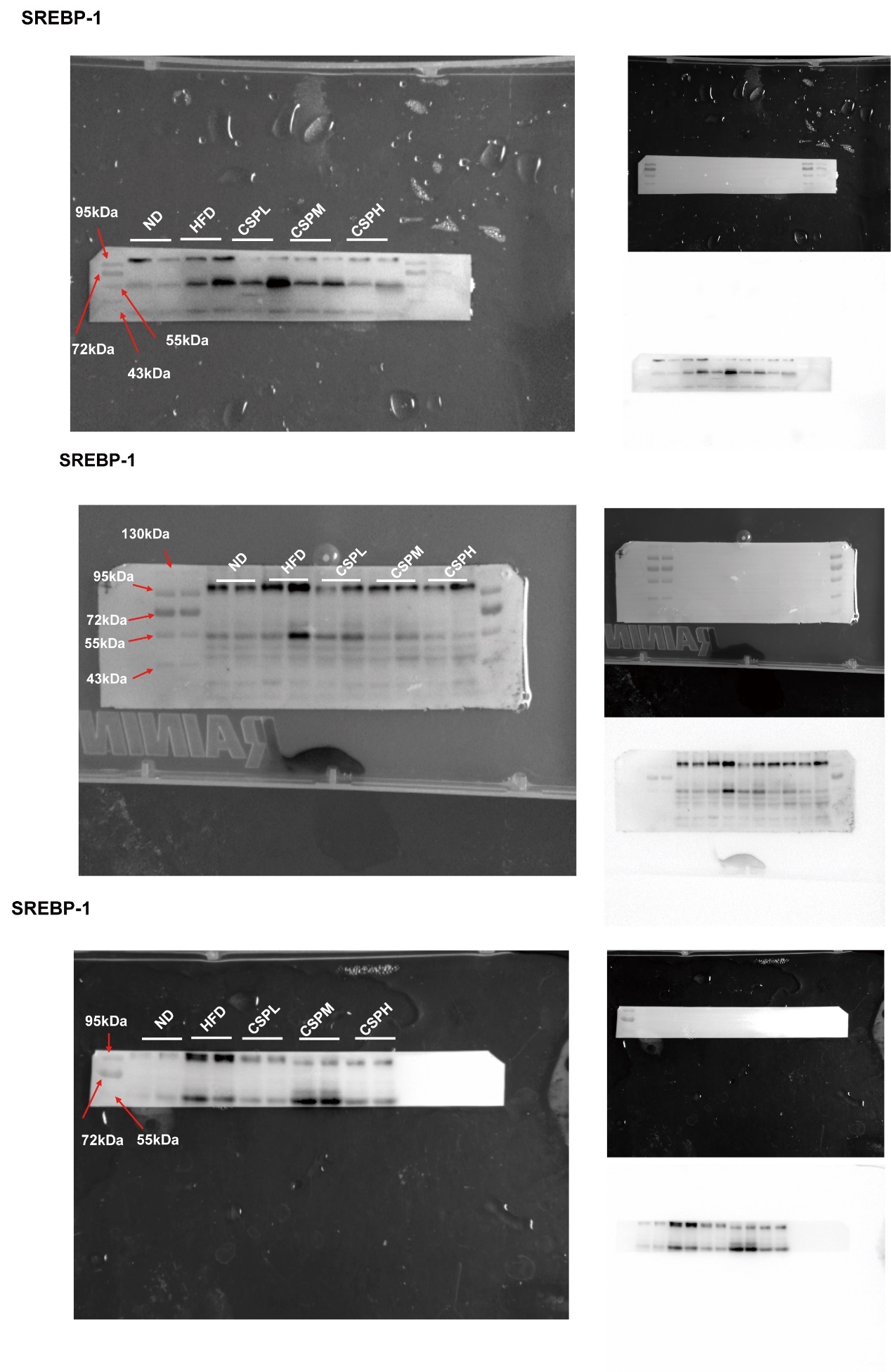


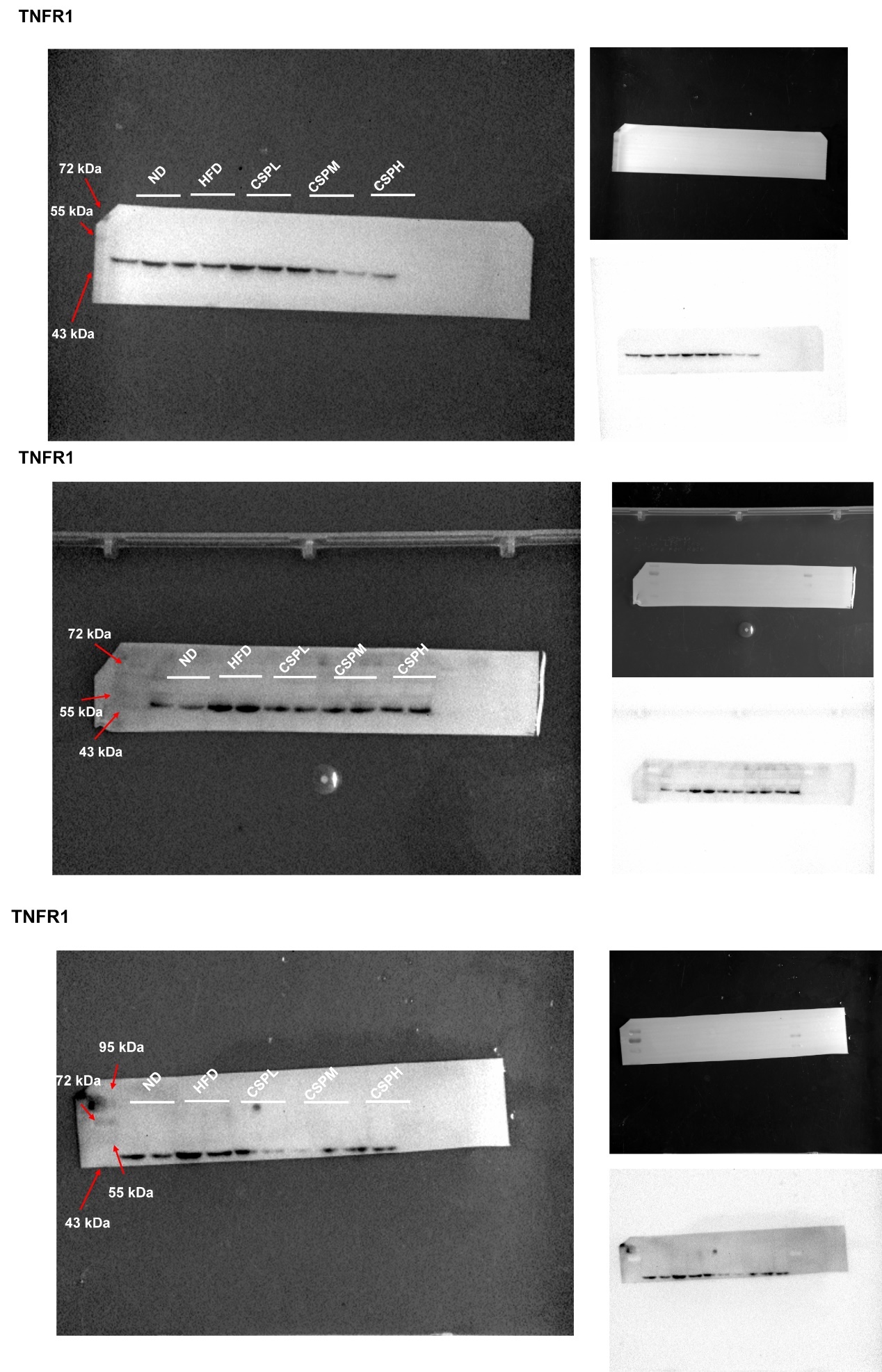


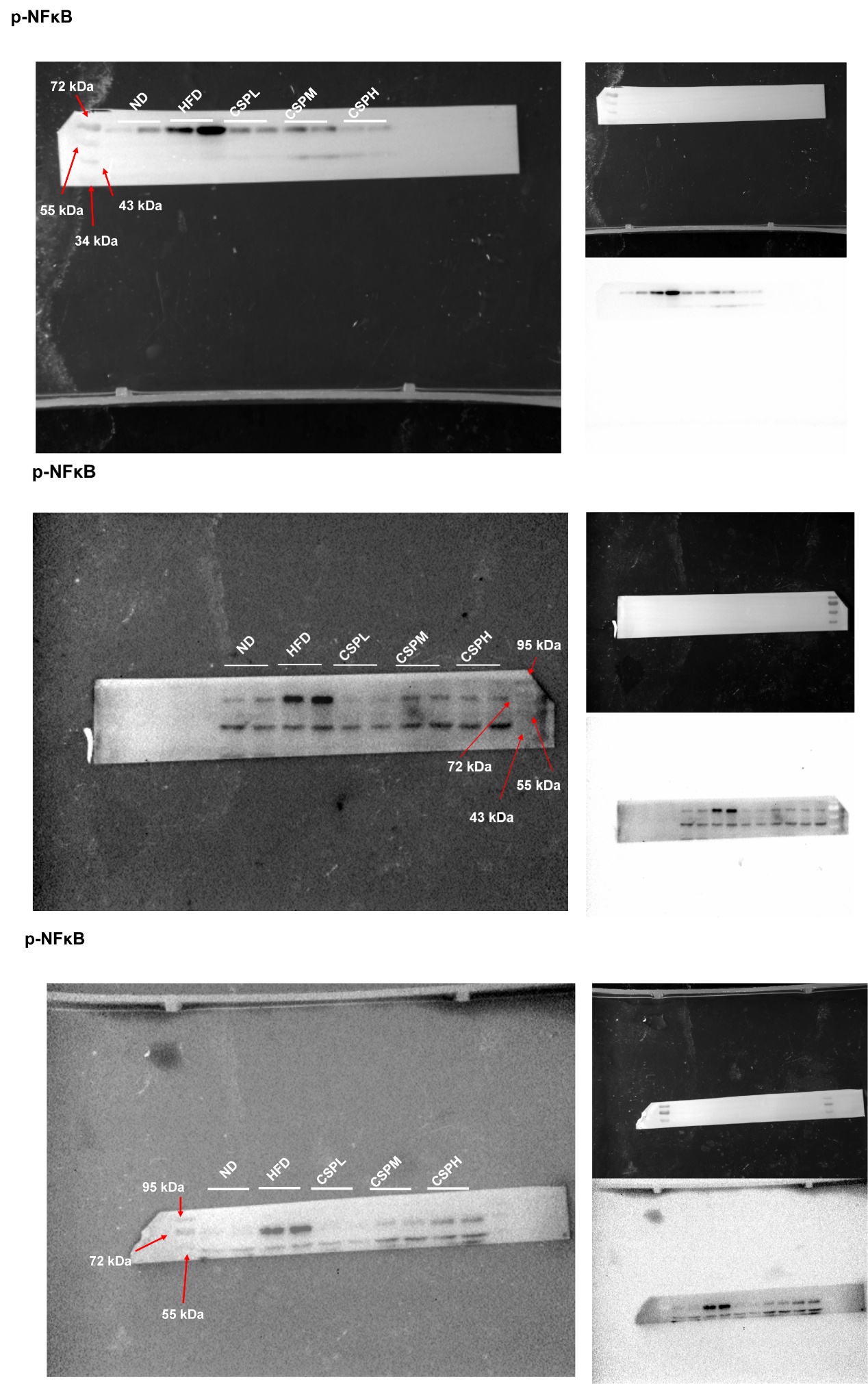


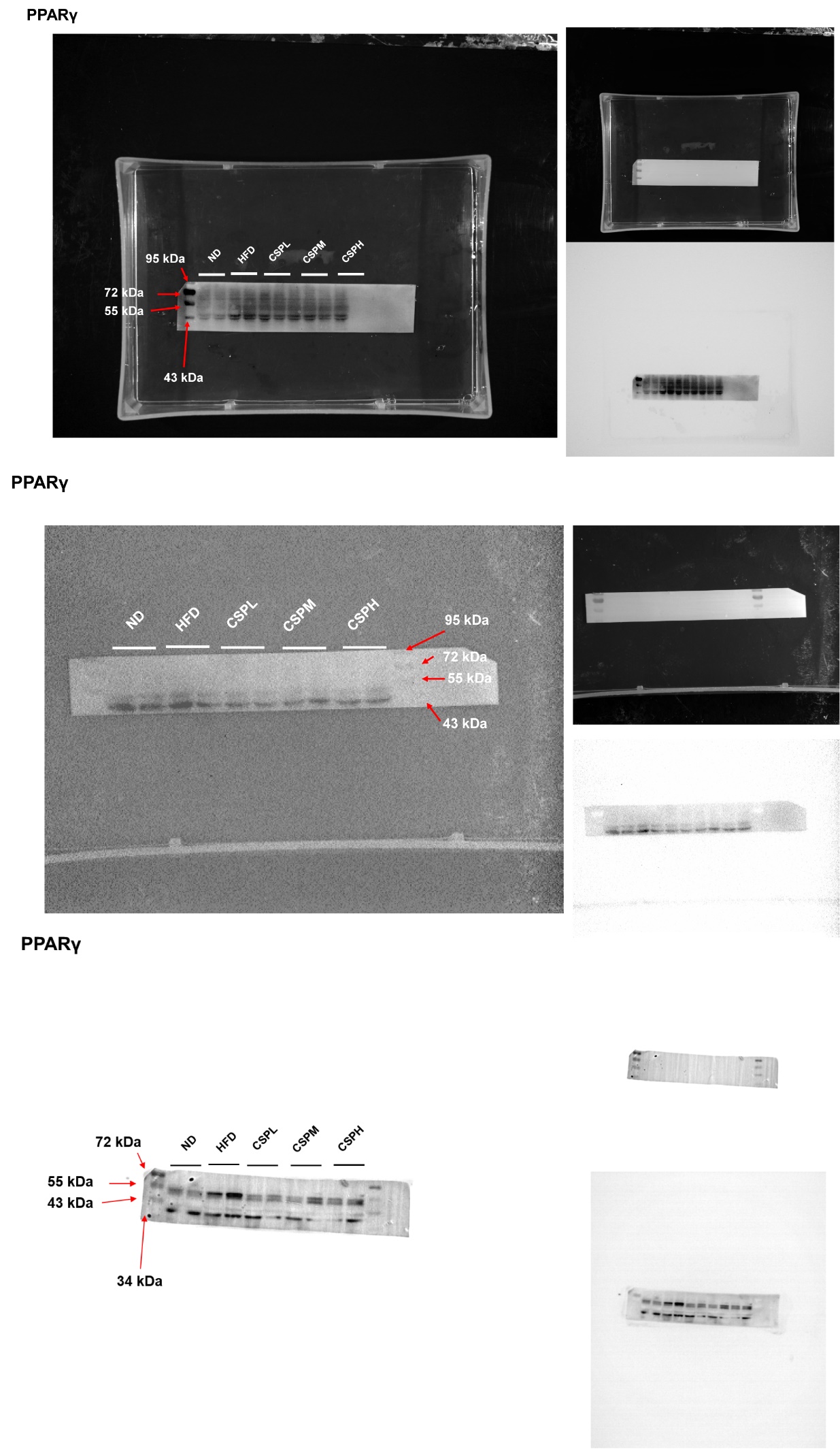


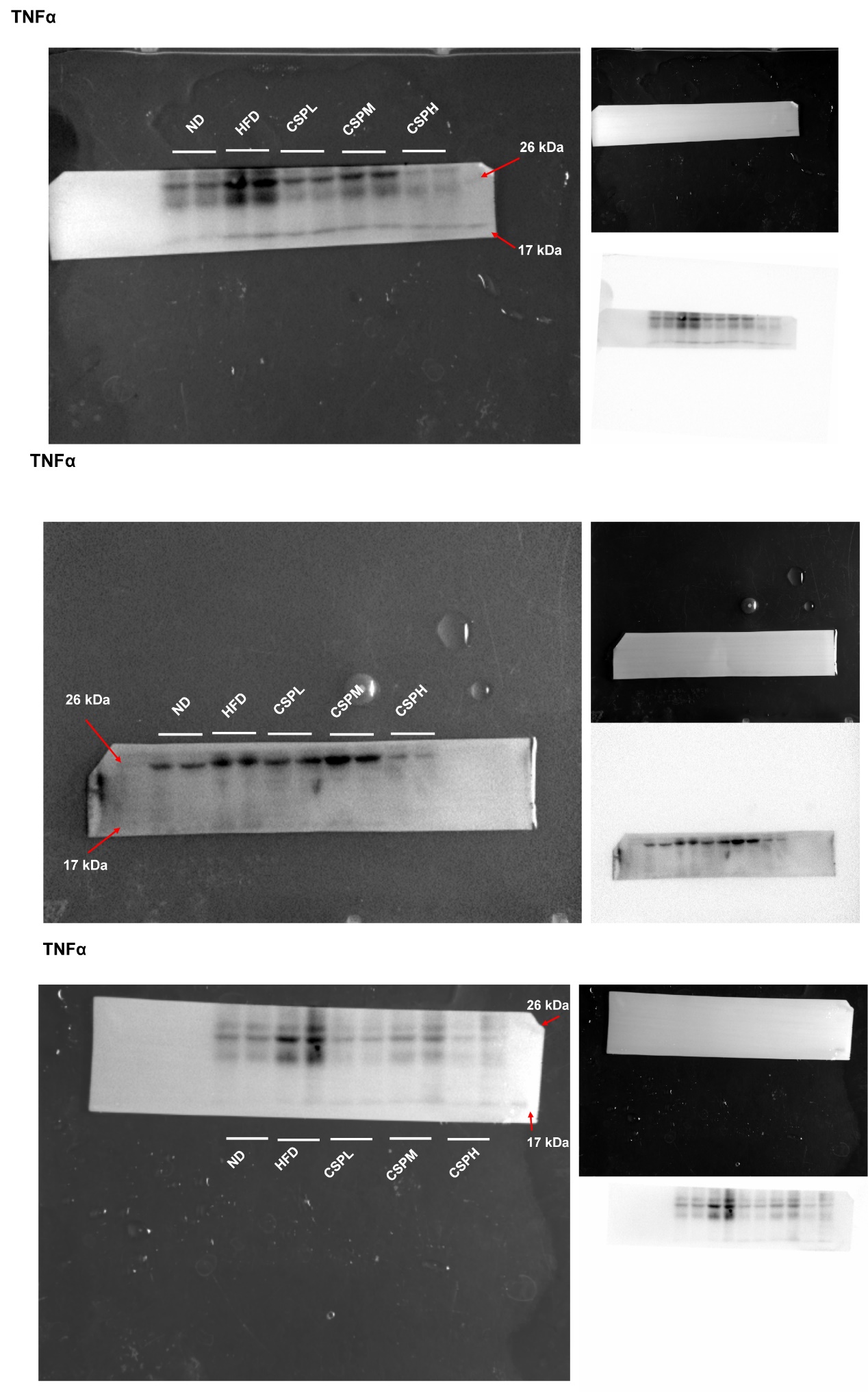


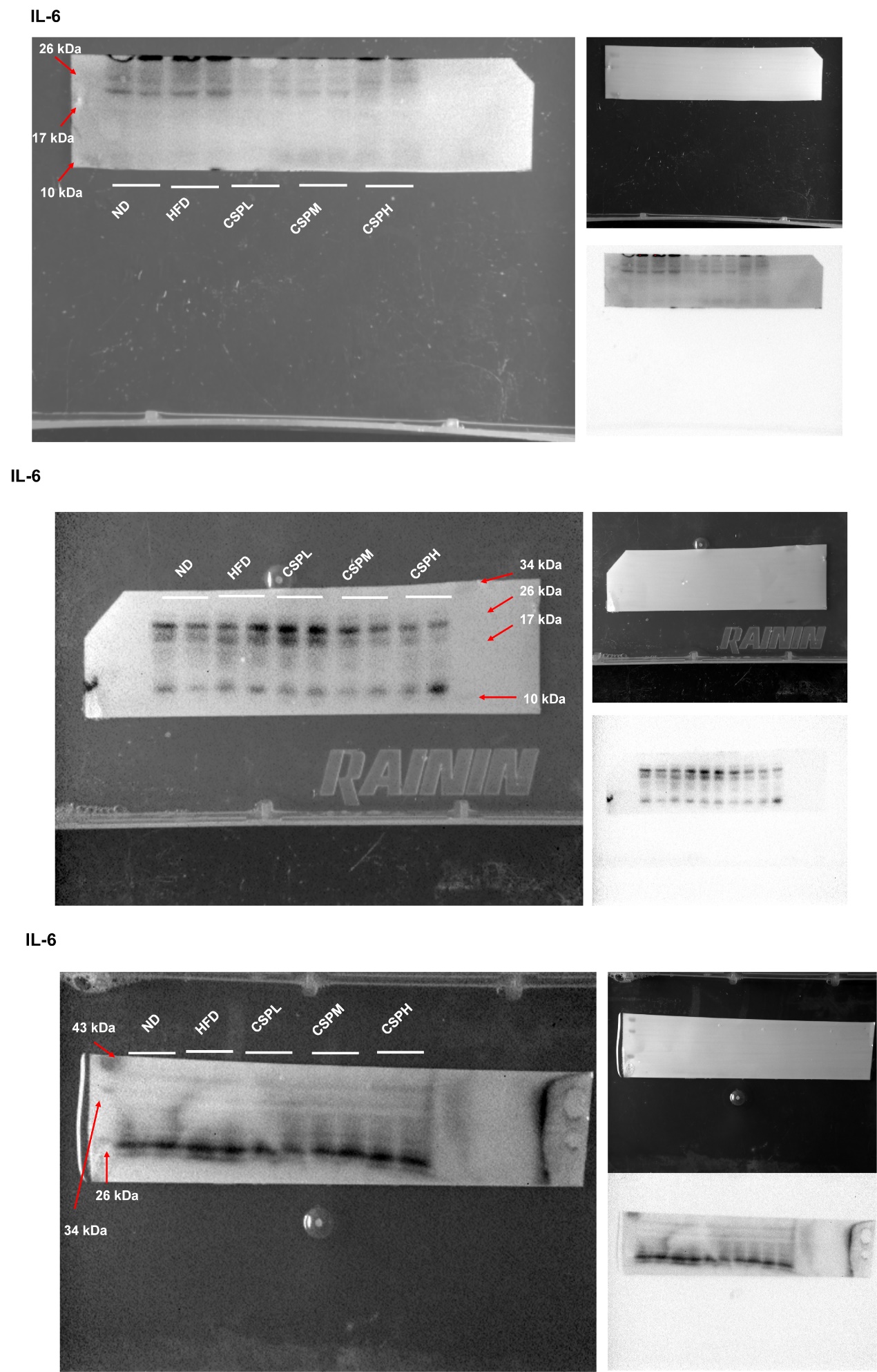


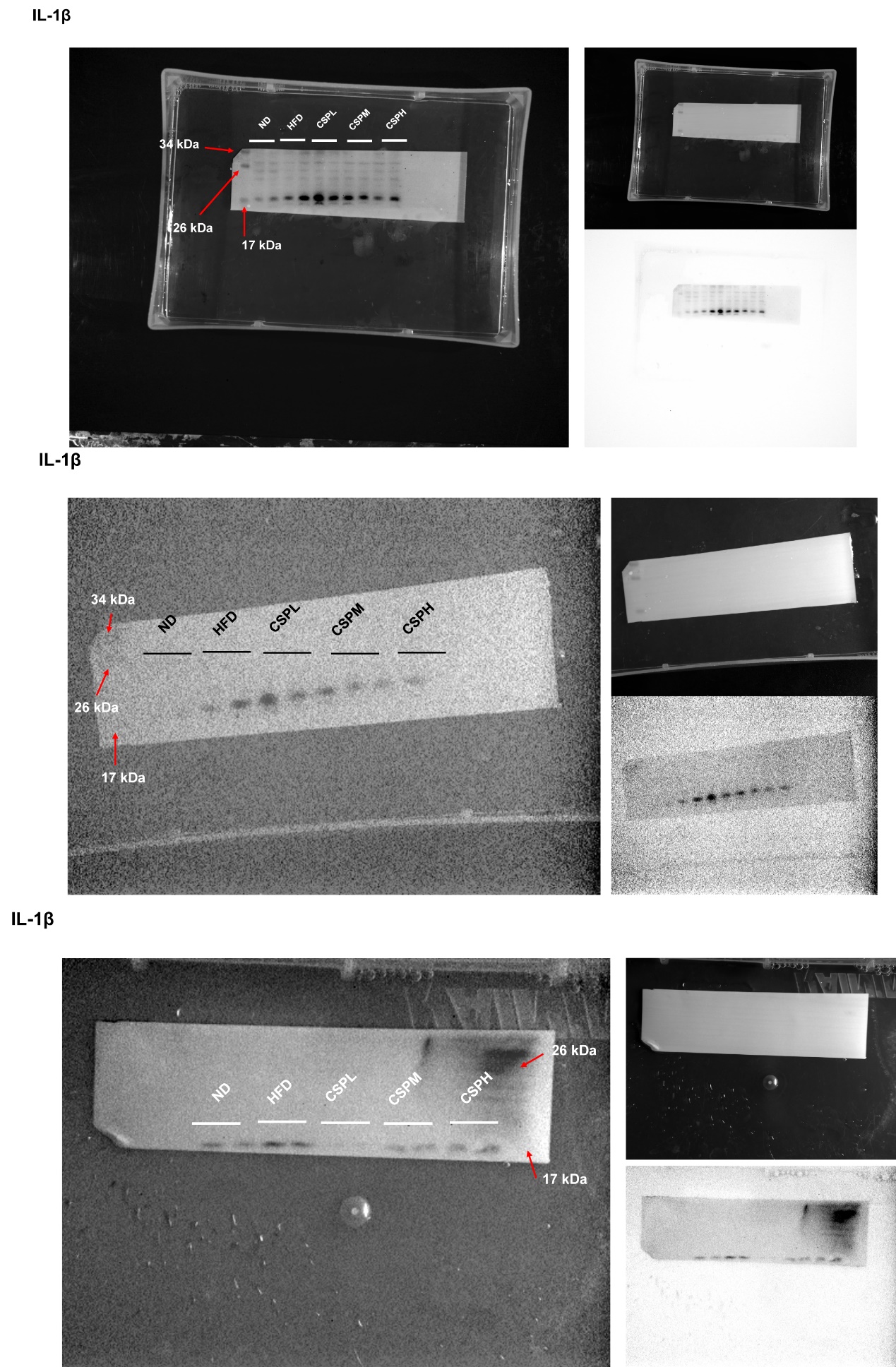


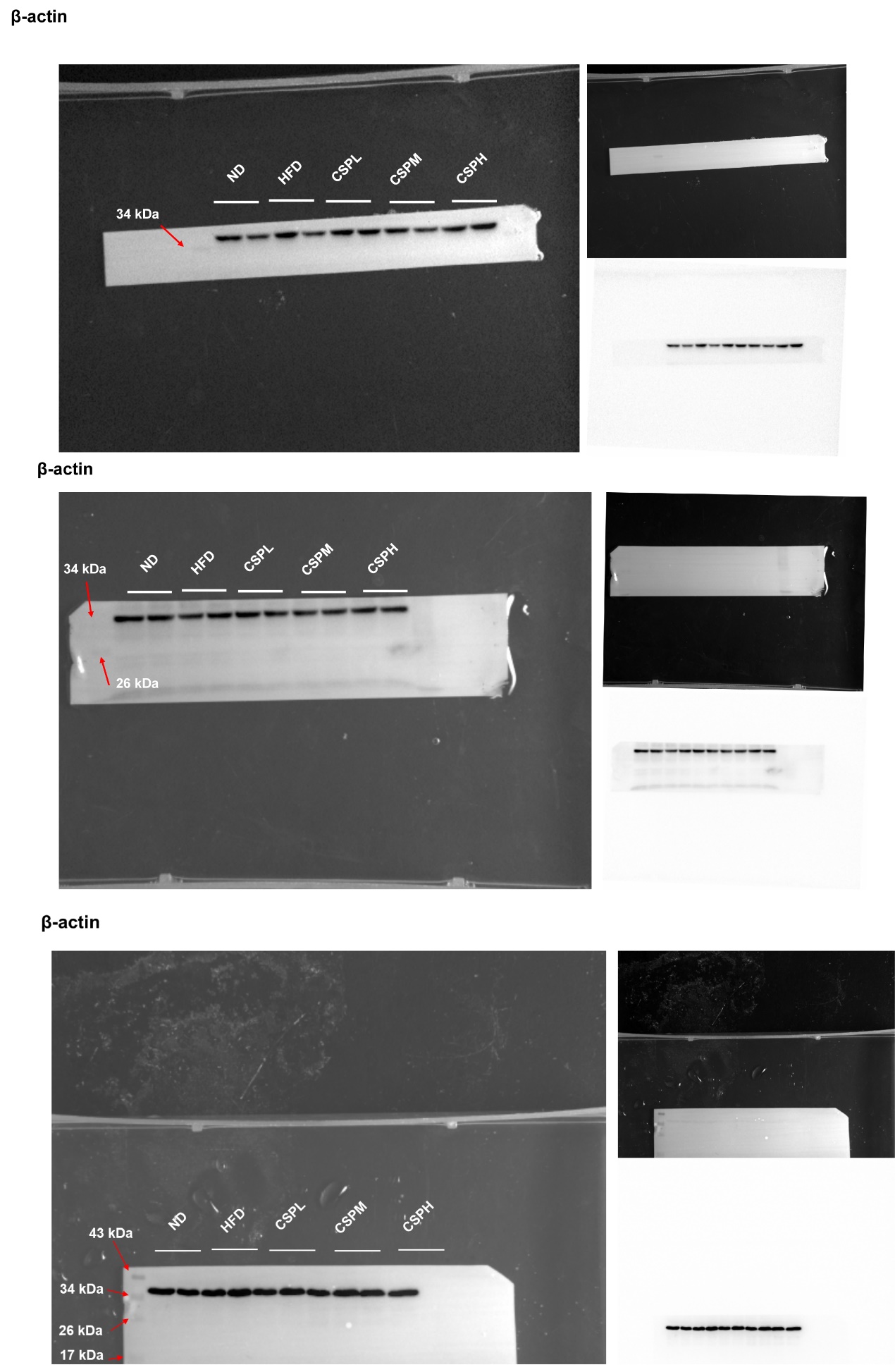


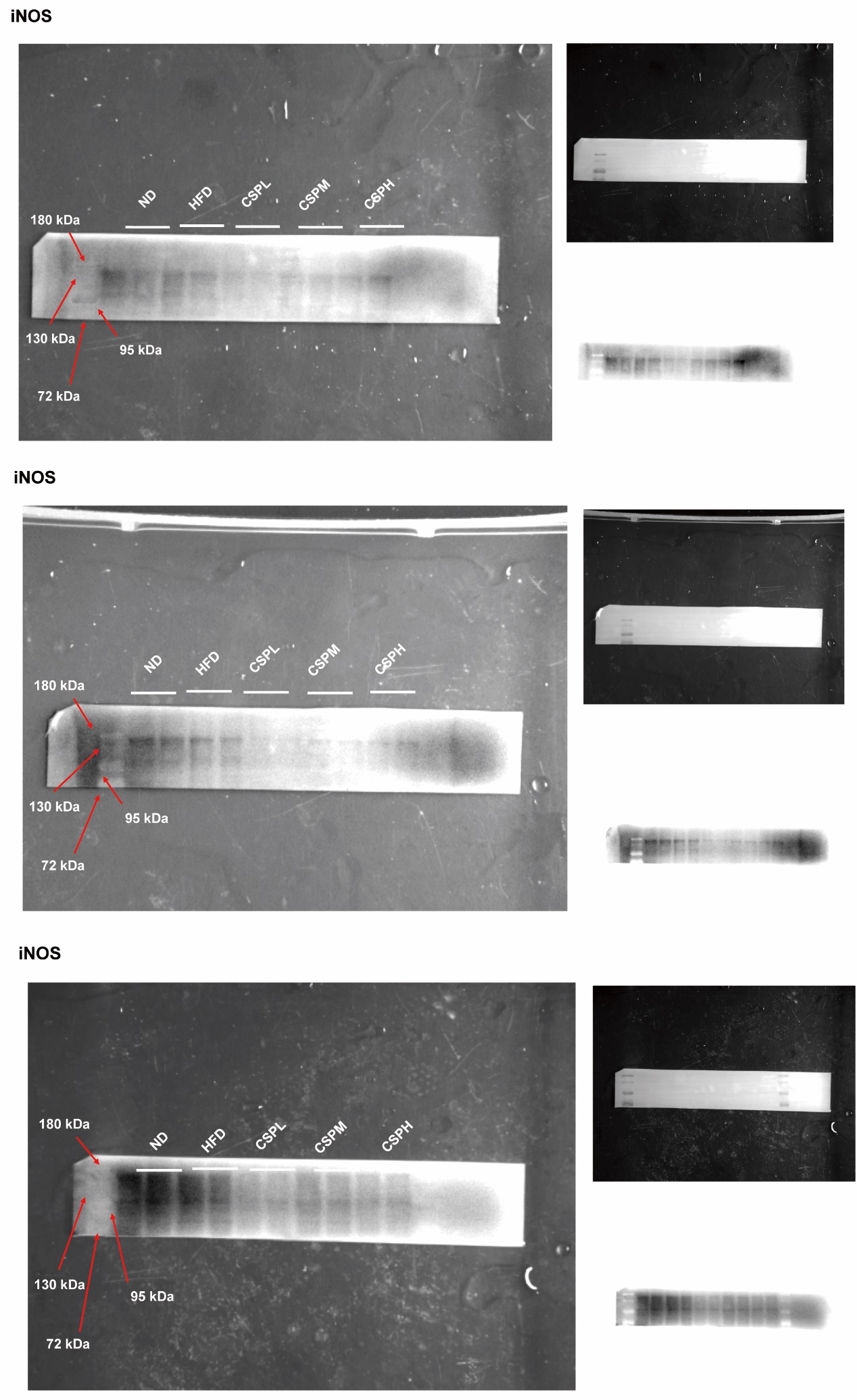

Supplement: Supplementary file 1 [file DataSheet3.docx]
