# Supplementary material for: Chaihu Shugan powder alleviates liver inflammation and hepatic steatosis in NAFLD mice: A network pharmacology study and in vivo experimental validation
Source: Front Pharmacol. 2022 Sep 12;13:967623. doi: 10.3389/fphar.2022.967623 (PMC9512055; doi:10.3389/fphar.2022.967623)
Supplement: Supplementary file 3 [file DataSheet2.docx]

**
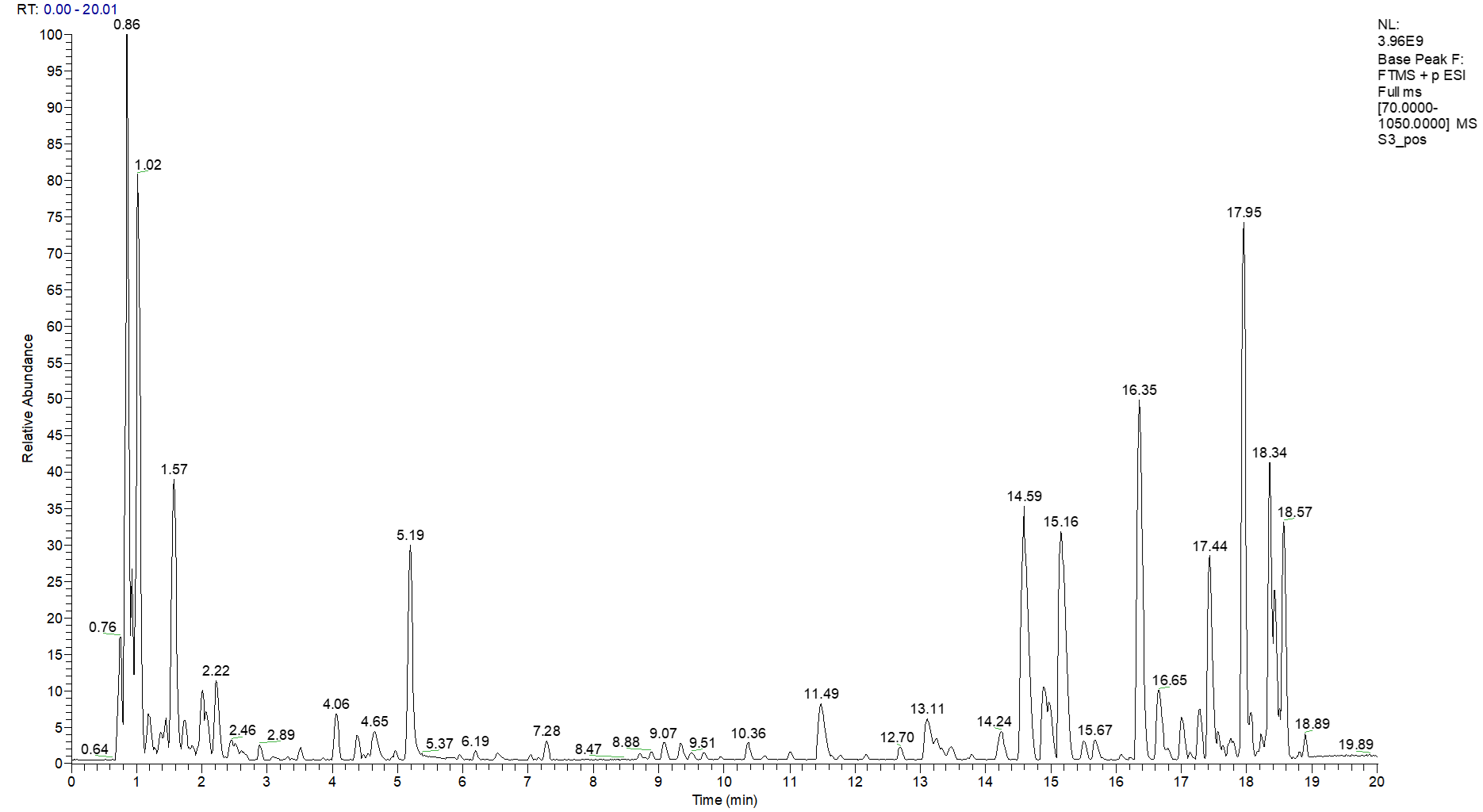
Supplementary material
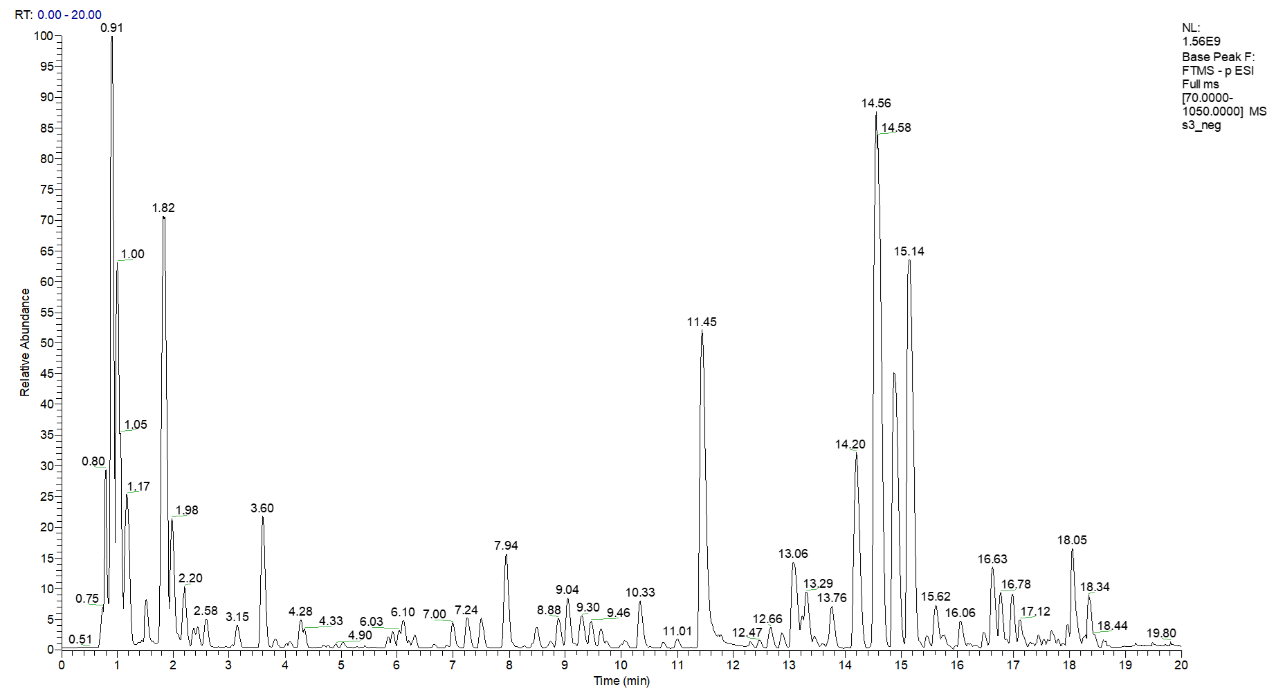
**

**Base peak ion (BPI) chromatogram in negative ion mode**

**Base peak ion (BPI) chromatogram in positive ion mode**

**Figure S1|** Total ion chromatogram (TIC) of CSP in positive and negative ion mode.

**Figure S2**| CSP decreased the FPG levels in mice with HFHFD plus CIS
